# Prevalence of Five Health-Related Behaviors for Chronic Disease Prevention Among Sexual and Gender Minority Adults — 25 U.S. States and Guam, 2016

**DOI:** 10.15585/mmwr.mm6732a4

**Published:** 2018-08-17

**Authors:** Timothy J. Cunningham, Fang Xu, Machell Town

**Affiliations:** 1 Division of Population Health, National Center for Chronic Disease Prevention and Health Promotion, CDC.

## Abstract

In recent decades, public health awareness of health disparities among lesbian, gay, bisexual, and transgender (LGBT) populations has increased (*1*). *Healthy People 2020* included objectives to improve health of LGBT persons.^†^ Five key health-related behaviors were found to be likely associated with reduced all-cause mortality: never smoking, performing regular physical activity, consuming no or moderate amounts of alcohol, having a normal body weight, and obtaining sufficient sleep daily (*2*). CDC estimated these five health-related behaviors among adults aged ≥21 years by sexual orientation and transgender status using data from the 2016 Behavioral Risk Factor Surveillance System (BRFSS) in 25 U.S. states and Guam. Patterns of these five health-related behaviors varied by sexual orientation among men and women, and among transgender adults. Lesbian and bisexual women were less likely to engage in all five health-related behaviors than were heterosexual women (5.4% and 6.9%, respectively, versus 10.6%). Compared with cisgender^§^ adults, male-to-female transgender adults were less likely to engage in any two of five health-related behaviors (12.3% versus 18.6%). Male-to-female transgender adults, however, were more likely to engage in any three of five health-related behaviors than were female-to-male transgender adults (47.2% versus 28.2%). The number of health-related behaviors did not differ between gay or bisexual men and heterosexual men. Continued efforts are needed to target LGBT populations for overall well-being, including strategies for health promotion and engagement in health-related behaviors.

BRFSS is an annual state-based, random-digit–dialed telephone survey of noninstitutionalized U.S. adults aged ≥18 years, which collects information on health-related topics.^¶^ In 2016, the median response rate of the combined landline and cellular telephone surveys from the 25 states** and Guam that participated in the sexual orientation and gender identity module was 42.8%.^††^ Based on the self-reported responses of adults aged ≥21 years,^§§^ sexual orientation was defined as being straight (heterosexual), lesbian or gay, bisexual, and other, and gender identity was defined as being not transgender (cisgender), transgender male-to-female, transgender female-to-male, and transgender nonconforming.

The prevalence and 95% confidence intervals of demographic characteristics (age, race/ethnicity, education, marital status, current employment status, household income, and home ownership) and of engaging in the five health-related behaviors was estimated by sexual orientation status for men and women separately, and by transgender status. The health-related behaviors^¶¶^ included 1) not currently cigarette smoking, 2) moderate or no drinking, 3) having a normal body weight, 4) engaging in any leisure-time physical activity, and 5) sleeping ≥7 hours, on average, during a 24-hour period. The number of the five health-related behaviors reported were categorized into five groups (0/1, 2, 3, 4, or 5). Because of small sample sizes, some categories of demographic characteristics and counts of health-related behaviors were collapsed when analyzing transgender status. Chi-squared tests were used to compare an overall difference for nominal variables and to test a trend difference for ordinal variables by sexual orientation in men and women, and by transgender status. Pairwise tests with linear contrasts were used to assess group differences with statistical significance set at p<0.05. Statistical software that accounts for survey weights and complex survey designs was used to conduct all analyses. All comparisons presented were statistically significant.

In 2016, among 86,185 men who answered the sexual orientation question, 92.7% reported being heterosexual, 2.2% reported being gay, and 1.5% reported being bisexual; among 114,842 women, 91.7% reported being heterosexual, 1.3% reported being lesbian, and 2.3% reported being bisexual (Table 1). Overall, sexual minority adults were younger than their heterosexual counterparts. Being a college graduate was more prevalent among gay men (42.0%) than among heterosexual men (27.9%) and bisexual men (23.3%). Among women, having a household income ≥$75,000 was less prevalent among bisexual women (18.9%) than among lesbian women (33.4%) and heterosexual women (27.4%), and being currently unemployed was more prevalent among bisexual women (35.3%) than among lesbian women (26.6%) and heterosexual women (28.4%). Overall, being single and renting a home were more prevalent among sexual minority adults.

**TABLE 1:** Distribution of demographics and health-related behaviors, among adults aged ≥21 years, by sexual orientation* — Behavioral Risk Factor Surveillance System, 25 U.S. states and Guam, 2016

Characteristic	No. (%)	Male^†^ (n = 86,185)				Female^§^ (n = 114,842)			
		Straight	Gay	Bisexual	Other/Don’t know/Refused	Straight	Lesbian	Bisexual	Other/Don’t know/Refused
		(N = 80,987)	(N = 1,748)	(N = 1,139)	(N = 2,311)	(N = 107,599)	(N = 1,190)	(N = 1,969)	(N = 4,084)
		% (95% CI)	% (95% CI)	% (95% CI)	% (95% CI)	% (95% CI)	% (95% CI)	% (95% CI)	% (95% CI)
**Overall**	201,027 (100)	92.7 (92.3−93.0)	2.2 (2.0−2.4)	1.5 (1.4−1.7)	3.7 (3.4−4.0)	91.7 (91.3−92.0)	1.3 (1.1−1.4)	2.3 (2.1−2.5)	4.8 (4.5−5.1)
**Age group (yrs)**									
21–24	6,304 (6.9)	6.8 (6.5−7.2)	11.6 (8.6−15.5)	13.5 (10.4−17.2)	4.8 (3.2−7.1)	6.4 (6.0−6.8)	12.2 (7.3−19.7)	23.4 (20.1−27.0)	5.4 (3.9−7.6)
25–34	19,092 (17.1)	17.6 (17.0−18.1)	24.8 (21.1−28.8)	29.6 (24.1−35.6)	16.0 (12.8−19.7)	15.8 (15.3−16.3)	22.5 (17.8−28.0)	35.9 (32.2−39.7)	15.5 (13.0−18.3)
35–44	22,712 (17.1)	17.6 (17.1−18.1)	13.8 (11.2−17.0)	15.1 (11.7−19.3)	20.4 (17.0−24.4)	16.5 (16.1−17.0)	16.9 (12.2−23.1)	18.1 (15.2−21.4)	18.7 (15.9−21.9)
45–54	32,514 (18.4)	18.8 (18.3−19.4)	21.8 (18.5−25.5)	14.7 (11.6−18.4)	16.0 (13.3−19.2)	18.3 (17.8−18.8)	21.1 (16.8−26.2)	10.5 (8.4−13.0)	16.1 (13.7−18.9)
55–64	45,703 (18.5)	18.8 (18.3−19.3)	17.1 (13.7−21.2)	14.5 (11.4−18.3)	18.4 (15.2−22.2)	18.7 (18.3−19.2)	13.8 (11.1−16.9)	6.0 (4.7−7.6)	16.3 (13.5−19.7)
≥65	72,269 (22.1)	20.3 (19.9−20.8)	10.8 (8.7−13.4)	12.7 (9.8−16.4)	24.3 (20.9−28.1)	24.3 (23.8−24.8)	13.5 (8.4−20.8)	6.2 (4.9−7.9)	27.9 (25.2−30.7)
**Race/Ethnicity**									
White, non-Hispanic	155,778 (63.1)	64.7 (64.0−65.4)	66.1 (61.2−70.7)	59.5 (53.8−64.9)	27.3 (24.2−30.7)	64.6 (63.9−65.2)	58.4 (50.9−65.5)	65.9 (61.9−69.6)	28.7 (26.0−31.5)
Black, non-Hispanic	13,884 (10.6)	9.8 (9.4−10.2)	8.9 (6.5−12.1)	14.2 (10.6−18.7)	6.4 (4.7−8.6)	11.6 (11.2−12.1)	13.5 (9.9−18.1)	10.0 (7.9−12.7)	7.2 (5.8−9.0)
Hispanic	13,968 (17.6)	16.8 (16.2−17.5)	14.2 (11.3−17.8)	17.3 (13.2−22.4)	51.5 (47.0−56.0)	15.9 (15.4−16.5)	16.7 (11.2−24.1)	14.6 (11.7−18.0)	47.6 (44.0−51.3)
Other, non-Hispanic	9,459 (7.2)	7.2 (6.8−7.6)	9.0 (5.8−13.9)	7.0 (4.8−9.9)	14.3 (10.9−18.5)	6.6 (6.1−7.0)	—^¶^	5.7 (4.0−8.0)	15.9 (12.6−19.8)
Multiracial	4,618 (1.4)	1.5 (1.3−1.7)	1.7 (1.1−2.7)	—^¶^	—^¶^	1.3 (1.2−1.5)	—^¶^	3.9 (2.8−5.3)	0.6 (0.4−0.9)
**Education**									
Less than HS	15,216 (14.8)	14.1 (13.5−14.7)	5.0 (3.2−7.7)	14.9 (11.2−19.7)	51.7 (47.4−56.1)	12.8 (12.4−13.4)	12.0 (7.8−18.0)	10.8 (8.4−13.9)	45.9 (42.4−49.5)
HS diploma or GED	54,997 (27.0)	28.9 (28.3−29.5)	20.8 (16.7−25.5)	26.6 (22.3−31.5)	20.4 (17.4−23.8)	25.9 (25.4−26.4)	20.7 (15.4−27.3)	23.9 (20.9−27.2)	25.7 (22.6−29.0)
Some college	53,257 (30.5)	29.1 (28.5−29.8)	32.2 (28.1−36.7)	35.2 (29.7−41.0)	14.6 (11.8−17.8)	32.7 (32.1−33.3)	34.2 (27.6−41.5)	39.3 (35.4−43.4)	16.6 (14.2−19.2)
College graduate	76,931 (27.7)	27.9 (27.4−28.4)	42.0 (37.8−46.2)	23.3 (19.7−27.3)	13.3 (11.3−15.6)	28.6 (28.1−29.1)	33.0 (27.9−38.7)	25.9 (23.1−29.0)	11.8 (10.2−13.7)
**Marital status**									
Married	106,354 (54.3)	57.6 (56.9−58.3)	19.5 (16.5−22.9)	30.9 (26.1−36.1)	59.1 (54.8−63.1)	53.4 (52.8−54.1)	30.1 (24.7−36.1)	29.2 (25.9−32.8)	47.4 (43.8−51.1)
Single**	34,844 (24.4)	25.9 (25.2−26.5)	71.5 (67.6−75.1)	54.5 (49.1−59.9)	24.9 (21.5−28.6)	20.6 (20.1−21.2)	52.8 (45.9−59.6)	51.3 (47.4−55.2)	21.3 (18.6−24.2)
Others	58,698 (21.4)	16.5 (16.1−17.0)	9.0 (6.8−11.7)	14.6 (11.6−18.2)	16.1 (13.5−19.0)	26.0 (25.4−26.5)	17.1 (11.6−24.4)	19.5 (16.5−22.8)	31.3 (28.4−34.4)
**Employment** ^††^									
Currently employed	99,608 (58.2)	66.8 (66.2−67.4)	66.3 (61.8−70.6)	61.8 (56.2−67.1)	64.7 (60.7−68.5)	50.9 (50.2−51.5)	60.3 (53.1−67.0)	59.9 (56.0−63.7)	35.4 (32.1−38.9)
Not currently employed	36,923 (22.2)	14.1 (13.6−14.6)	20.3 (17.0−24.1)	25.2 (20.5−30.6)	15.2 (12.6−18.2)	28.4 (27.8−29.0)	26.6 (21.1−33.0)	35.3 (31.6−39.1)	44.7 (41.1−48.3)
Retired	63,211 (19.6)	19.1 (18.6−19.6)	13.4 (10.1−17.5)	13.0 (9.9−16.9)	20.1 (17.2−23.3)	20.7 (20.3−21.2)	13.1 (8.2−20.4)	4.8 (3.6−6.3)	19.9 (17.7−22.3)
**Household income ($)**									
<25,000	44,739 (23.3)	19.6 (19.0−20.1)	22.6 (19.2−26.4)	29.0 (24.4−34.1)	40.9 (36.7−45.2)	24.6 (24.1−25.2)	27.3 (21.4−34.2)	33.6 (30.0−37.5)	43.2 (39.8−46.7)
≥25,000−34,999	18,289 (8.8)	8.7 (8.3−9.1)	5.9 (4.4−7.9)	14.5 (10.7−19.3)	11.5 (8.9−14.8)	9.0 (8.6−9.3)	7.4 (4.9−11.1)	8.3 (6.6−10.3)	6.5 (5.3−7.9)
≥35,000−50,000	24,742 (11.7)	12.4 (11.9−12.8)	14.2 (10.4−19.2)	11.7 (8.8−15.3)	7.9 (5.8−10.7)	11.4 (11.0−11.8)	9.3 (6.9−12.3)	12.5 (9.9−15.8)	6.7 (4.9−8.9)
≥50,000−74,999	28,138 (13.0)	14.1 (13.7−14.6)	14.9 (12.1−18.1)	7.2 (5.4−9.5)	6.8 (4.7−9.8)	12.8 (12.4−13.3)	12.5 (9.1−16.9)	11.2 (8.9−14.0)	3.2 (2.1−4.9)
≥$75,000	57,018 (29.5)	34.2 (33.6−34.8)	34.2 (30.3−38.3)	24.1 (19.6−29.3)	9.3 (7.5−11.5)	27.4 (26.9−28.0)	33.4 (26.9−40.7)	18.9 (16.2−22.0)	5.4 (4.2−6.9)
Don't know/ Refused	28,101 (13.7)	11.1 (10.7−11.5)	8.2 (6.2−10.8)	13.6 (10.0−18.1)	23.5 (20.4−27.0)	14.7 (14.3−15.2)	10.1 (7.1−14.1)	15.4 (12.9−18.4)	35.1 (31.7−38.5)
**Home ownership**									
Own	145,421 (72.5)	73.3 (72.7−73.8)	60.7 (56.3−64.9)	56.2 (50.5−61.8)	55.8 (51.4−60.2)	74.2 (73.7−74.8)	67.4 (61.4−72.9)	46.8 (42.7−50.9)	58.0 (54.5−61.4)
Rent	47,743 (27.5)	26.7 (26.2−27.3)	39.3 (35.1−43.7)	43.8 (38.2−49.5)	44.2 (39.8−48.6)	25.8 (25.2−26.3)	32.6 (27.1−38.6)	53.2 (49.1−57.3)	42.0 (38.6−45.5)
**Health-related behaviors** ^§§^									
No current cigarette smoking	169,483 (83.7)	81.4 (80.9−81.9)	77.0 (73.0−80.6)	77.1 (72.3−81.2)	84.4 (80.8−87.4)	86.0 (85.6−86.4)	75.0 (68.6−80.4)	71.7 (68.2−75.0)	94.3 (92.7−95.5)
Moderate or no drinking	131,609 (63.8)	60.8 (60.1−61.5)	51.8 (47.3−56.3)	59.1 (53.7−64.3)	71.1 (66.6−75.1)	66.2 (65.6−66.8)	50.6 (43.7−57.5)	47.1 (43.2−51.1)	86.1 (83.6−88.3)
Having a normal weight	58,649 (31.2)	25.0 (24.4−25.6)	40.3 (35.7−45.1)	29.8 (25.3−34.8)	25.2 (21.8−29.1)	37.0 (36.4−37.6)	30.4 (25.0−36.4)	35.8 (32.0−39.8)	36.4 (32.5−40.6)
Any leisure-time physical activity	150,477 (75.3)	77.9 (77.4−78.5)	82.0 (78.4−85.0)	78.1 (73.2−82.3)	63.2 (58.8−67.4)	73.8 (73.3−74.4)	80.6 (75.7−84.7)	77.6 (74.2−80.7)	59.5 (56.0−63.0)
Sleeping ≥7 hours/24-hour period	134,453 (65.0)	64.2 (63.6−64.9)	63.9 (59.1−68.4)	59.3 (53.8−64.6)	68.2 (63.8−72.2)	65.9 (65.3−66.5)	55.3 (48.0−62.3)	56.1 (52.2−60.0)	67.2 (63.8−70.5)
**No. of health-related behaviors**									
0 and 1	9,163 (5.8)	6.6 (6.3−7.0)	6.5 (4.6−9.2)	8.5 (5.9−12.2)	6.1 (4.3−8.4)	4.9 (4.6−5.2)	10.0 (6.5−15.0)	10.7 (8.5−13.6)	2.6 (1.6−4.2)
2	31,368 (18.6)	20.0 (19.5−20.6)	17.9 (15.0−21.2)	20.4 (16.3−25.3)	20.1 (15.7−25.3)	17.2 (16.7−17.7)	23.0 (16.7−30.8)	26.7 (23.0−30.8)	11.7 (9.8−14.1)
3	64,600 (35.2)	36.7 (36.1−37.4)	37.2 (33.0−41.7)	36.2 (31.1−41.6)	36.5 (31.9−41.4)	33.7 (33.1−34.3)	37.2 (30.3−44.6)	32.5 (28.8−36.4)	35.6 (31.7−39.7)
4	60,702 (31.7)	29.8 (29.2−30.5)	30.1 (25.6−35.0)	26.7 (21.8−32.1)	29.9 (25.9−34.2)	33.7 (33.1−34.3)	24.4 (19.7−29.9)	23.2 (19.9−26.8)	38.7 (34.4−43.1)
5	17,696 (8.7)	6.8 (6.5−7.2)	8.2 (6.2−10.9)	8.3 (6.0−11.3)	7.4 (5.7−9.6)	10.6 (10.1−11.0)	5.4 (3.5−8.1)	6.9 (5.2−9.0)	11.4 (9.2−14.0)

**Abbreviations**: CI = confidence interval; GED = general educational development high school equivalency diploma; HS = high school.

* Sexual orientation is based on responses to a question, “Do you consider yourself to be straight, lesbian, gay, bisexual, other, or don’t know/not sure?”

^†^ Significant associations (p<0.05) between status of sexual orientation and characteristics included age, education, household income (Cochran-Mantel-Haenszel chi-squared for trends), marital status, employment, home ownership, not currently smoking cigarettes, moderate or no drinking, and having a normal weight (Cochran-Mantel-Haenszel chi-squared for general association). Other/Don’t know/Refused was not included in the test.

^§^ Significant associations (p<0.05) between status of sexual orientation and characteristics included age, household income, number of health-related behaviors (Cochran-Mantel-Haenszel chi-squared for trends), race/ethnicity, marital status, employment, home ownership, not currently smoking cigarettes, moderate or no drinking, any leisure-time exercise, and sufficient sleep (≥7 hours) (Cochran-Mantel-Haenszel chi-squared for general association). Other/Don’t know/Refused was not included in the comparison.

^¶^ Data are suppressed if relative standard error >0.3 or sample size <50. Relative standard error was calculated as a ratio of standard error and mean of the estimate.

** Single includes those who were never married or a member of an unmarried couple.

^††^ Employment status is defined as retired, and employed if responses are “currently employed for wages” or “currently self-employed,” “Not employed” included adults who were currently out of work, a homemaker, a student, or unable to work.

^§§^ Not a current cigarette smoker were respondents who reported not having smoked 100 cigarettes or more in their lifetime or having smoked at least 100 cigarettes in their lifetime but not smoking at the time of the survey. Moderate or no drinking in the past 30 days was defined as respondents’ self-reported no alcohol drinking or drinking ≤2 alcoholic drinks per day for men and ≤1 alcoholic drink per day for women, and not binge drinking or heavy drinking. Binge drinking was defined as drinking ≥5 drinks on one occasion for men and ≥4 drinks on one occasion for women. Heavy drinking was defined as drinking ≥15 drinks per week for men and ≥8 drinks per week for women in the past 30 days. Having a normal body weight was defined as body mass index ≥18.5 kg/m^2^ and <25 kg/m^2^. Any leisure-time physical activity was defined as an affirmative response to a question, “During the past month, other than your regular job, did you participate in any physical activities or exercises such as running, calisthenics, golf, gardening, or walking for exercise?” Sufficient sleep was defined ≥7 hours in response to the question, “On average, how many hours of sleep do you get in a 24-hour period?”

Compared with heterosexual men, gay men had a lower prevalence of not currently smoking cigarettes (77.0% versus 81.4%) and moderate or no drinking (51.8% versus 60.8%), but had a higher prevalence of performing any leisure-time exercise (82.0% versus 77.9%); gay men also had a higher prevalence of having a normal body weight (40.3%) than did bisexual (29.8%) and heterosexual men (25.0%). The prevalence of not currently smoking cigarettes, moderate or no alcohol consumption, and getting ≥7 hours’ sleep during a 24-hour period was higher among heterosexual women (86.0%, 66.2%, and 65.9%, respectively) than among lesbian (75.0%, 50.6%, and 55.3%, respectively) and bisexual women (71.7%, 47.1%, and 56.1%, respectively). Engaging in any leisure-time exercise was more prevalent among lesbian (80.6%) and bisexual women (77.6%) than among heterosexual women (73.8%); however, having a normal body weight was less prevalent among lesbian women (30.4%) than among heterosexual women (37.0%); the difference in prevalence between heterosexual women and bisexual women (35.8%) was not statistically significant. In addition, the prevalence of reporting zero or one health-related behavior was higher among lesbian (10.0%) and bisexual (10.7%) women than among heterosexual women (4.9%), and the prevalence of reporting all five health-related behaviors was lower among lesbian (5.4%) and bisexual (6.9%) women than among heterosexual women (10.6%) (Table 1).

Among 200,874 adults from the 25 states and Guam who answered the gender identity question, 98.3% reported being cisgender, 0.2% reported being male-to-female transgender, and 0.1% each reported being female-to-male transgender and transgender nonconforming (Table 2). Being a college graduate was more prevalent among cisgender adults (27.9%) than among transgender male-to-female adults (9.8%). Being single was more prevalent among transgender female-to-male (40.3%) and transgender nonconforming adults (55.4%) than cisgender adults (24.4%). The prevalence of having household income of <$25,000 and renting versus owning a home was higher among transgender adults than among cisgender adults.

**TABLE 2:** Distribution of demographics and health-related behaviors, among adults aged ≥21 years, by transgender status* — Behavioral Risk Factor Surveillance System, 25 U.S. states and Guam, 2016

Characteristic	No. (%)	Cisgender^†^	Transgender, male-to-female	Transgender, female-to-male	Transgender, nonconforming	Don’t know/Refused
		(N = 197,966)	(N = 344)	(N = 234)	(N = 167)	(N = 2,163)
		% (95% CI)	% (95% CI)	% (95% CI)	% (95% CI)	% (95% CI)
**Overall**	200,874 (100)	98.3 (98.1−98.4)	0.2 (0.2−0.3)	0.1 (0.1−0.2)	0.1 (0.1−0.1)	1.3 (1.2−1.4)
**Age group (yrs)** ^§^						
21–44	48,079 (41.1)	41.1 (40.7−41.6)	45.8 (33.5−58.6)	53.5 (41.6−65.0)	66.6 (54.8−76.6)	29.3 (25.0−34.0)
45–64	78,160 (36.8)	36.9 (36.4−37.3)	39.5 (26.9−53.7)	28.0 (19.2−38.0)	23.4 (15.1−34.5)	36.9 (32.0−42.2)
≥65	72,207 (22.1)	22.0 (21.7−22.3)	14.7 (9.4−22.4)	19.0 (12.9−27.0)	—^¶^	33.7 (29.5−38.3)
**Race/Ethnicity**						
White, non-Hispanic	155,668 (63.1)	63.5 (63.0−63.9)	50.2 (37.3−63.0)	56.4 (43.5−68.5)	46.8 (34.4−59.7)	36.1 (31.9−40.5)
Black, non-Hispanic	13,873 (10.6)	10.6 (10.3−10.9)	—^¶^	—^¶^	—^¶^	9.8 (7.5−12.7)
Hispanic	13,953 (17.6)	17.4 (17.0−17.8)	—^¶^	—^¶^	—^¶^	35.2 (30.3−40.4)
Other, non-Hispanic	14,070 (8.7)	8.5 (8.2−8.9)	—^¶^	—^¶^	—^¶^	18.9 (14.0−24.9)
**Education** ^§^						
Less than HS	15,192 (14.7)	14.4 (14.1−14.8)	32.8 (20.4−48.3)	—^¶^	—^¶^	36.8 (32.0−41.9)
HS diploma or GED	54,934 (27.0)	27.0 (26.6−27.3)	28.8 (20.3−39.0)	33.9 (24.1−45.5)	29.4 (18.1−43.9)	28.8 (24.3−33.7)
Some college	53,220 (30.5)	30.7 (30.2−31.1)	28.6 (18.0−42.3)	34.5 (23.0−48.1)	38.2 (26.2−51.8)	18.7 (15.3−22.7)
College graduate	76,905 (27.7)	27.9 (27.6−28.3)	9.8 (6.2−15.2)	—^¶^	19.8 (12.5−29.9)	15.7 (13.1−18.6)
Marital status**						
Married	106,277 (54.3)	54.3 (53.9−54.8)	47.7 (34.9−60.8)	33.0 (23.1−44.6)	27.4 (18.5−38.5)	55.0 (50.1−59.7)
Single^††^	34,824 (24.4)	24.4 (24.0−24.8)	34.3 (23.7−46.6)	40.3 (28.5−53.2)	55.4 (42.6−67.6)	18.1 (14.8−21.8)
Others	58,646 (21.4)	21.3 (20.9−21.6)	18.1 (12.1−26.1)	26.8 (17.9−38.0)	—^¶^	27.0 (23.1−31.2)
**Employment** ^§§^						
Currently employed	99,548 (58.2)	58.3 (57.9−58.8)	61.4 (49.7−72.0)	51.7 (39.6−63.6)	50.8 (37.7−63.7)	46.2 (41.3−51.2)
Not currently employed	36,894 (22.2)	22.1 (21.7−22.5)	24.2 (16.2−34.4)	30.9 (21.4−42.5)	—^¶^	27.8 (23.4−32.8)
Retired	63,149 (19.6)	19.6 (19.2−19.9)	14.4 (9.1−21.9)	17.3 (11.3−25.7)	—^¶^	26.0 (22.4−30.0)
**Household income ($)**						
<25,000	44,686 (23.3)	23.1 (22.8−23.5)	36.4 (25.9−48.2)	43.7 (32.0−56.2)	41.2 (28.6−55.0)	27.3 (23.5−31.3)
25,000−74,999	71,127 (33.5)	33.7 (33.3−34.1)	30.6 (18.3−46.4)	30.8 (21.5−41.9)	35.1 (23.7−48.4)	20.6 (16.7−25.2)
≥75,000	56,997 (29.5)	29.9 (29.5−30.3)	14.1 (8.9−21.7)	—^¶^	—^¶^	8.2 (6.3−10.7)
Don't know/Refused	28,064 (13.7)	13.3 (13.0−13.6)	—^¶^	—^¶^	—^¶^	43.9 (39.1−48.8)
Home ownership**						
Own	145,317 (72.5)	72.7 (72.3−73.0)	55.8 (42.7−68.1)	53.4 (41.0−65.4)	53.3 (39.0−67.0)	64.3 (59.5−68.7)
Rent	47,701 (27.5)	27.3 (27.0−27.7)	44.2 (31.9−57.3)	46.6 (34.6−59.0)	46.7 (33.0−61.0)	35.7 (31.3−40.5)
**Health-related behavior** ^¶¶^						
No current cigarette smoking	169,349 (83.7)	83.7 (83.4−84.0)	79.1 (68.5−86.8)	72.9 (59.1−83.3)	85.1 (75.2−91.6)	89.6 (86.6−92.0)
Moderate or no drinking	131,494 (63.8)	63.6 (63.2−64.0)	74.2 (62.7−83.1)	70.3 (57.9−80.4)	61.4 (47.7−73.4)	80.8 (76.0−84.8)
Having a normal weight	58,606 (31.2)	31.2 (30.7−31.6)	23.9 (15.7−34.5)	36.3 (23.6−51.4)	—^¶^	34.4 (29.0−40.2)
Any leisure-time physical activity	150,369 (75.3)	75.5 (75.1−75.9)	56.7 (42.9−69.6)	69.0 (57.7−78.4)	72.2 (57.1−83.5)	66.1 (61.4−70.5)
Sleeping ≥7 hours/24-hour period**	134,343 (65.0)	65.0 (64.6−65.5)	77.4 (68.3−84.5)	58.9 (46.2−70.5)	52.9 (39.7−65.7)	62.8 (57.7−67.6)
**No. of health-related behaviors**						
0 and 1	9,156 (5.8)	5.8 (5.6−6.0)	—^¶^	—^¶^	—^¶^	3.9 (2.3−6.3)
2	31,350 (18.6)	18.6 (18.3−19.0)	12.3 (7.9−18.6)	—^¶^	—^¶^	17.1 (12.3−23.2)
3	64,569 (35.2)	35.3 (34.8−35.7)	47.2 (33.5−61.4)	28.2 (19.0−39.7)	31.4 (20.4−44.9)	32.9 (27.7−38.5)
4 and 5	78,331 (40.3)	40.3 (39.9−40.8)	32.1 (21.8−44.5)	43.3 (30.1−57.5)	34.7 (23.2−48.4)	46.2 (40.2−52.4)

**Abbreviations:** CI = confidence interval; GED = general educational development high school equivalency diploma; HS = high school.

* Transgender status is based on responses of “Yes, transgender, male-to-female,” “Yes, transgender, female-to-male,” “Yes, transgender, gender nonconforming,” “No,” “Don’t know/not sure,” and “Refused” to a question, “Do you consider yourself to be transgender?”

^†^ Cisgender is related to a person whose gender identity corresponds with sex at birth.

^§^ Significant associations (p<0.05) between transgender status and characteristics based on Cochran-Mantel-Haenszel chi-squared for trends. Don’t know/Refused was not included in the test.

^¶^ Data are suppressed if relative standard error >0.3 or sample size <50. Relative standard error was calculated as a ratio of standard error and mean of the estimate.

** Significant associations (p<0.05) between transgender status and characteristics based on Cochran-Mantel-Haenszel chi-squared for general association. Don’t know/Refused was not included in the test.

^††^ Single includes those who were never married or a member of an unmarried couple.

^§§^ Employment status is defined as retired, and employed if responses are “currently employed for wages” or “currently self-employed,” “Not employed” included adults who were currently out of work, a homemaker, a student, or unable to work.

^¶¶^ Not a current cigarette smoker were respondents who reported not having smoked 100 cigarettes or more in their lifetime or having smoked at least 100 cigarettes in their lifetime but not smoking at the time of the survey. Moderate or no drinking in the past 30 days was defined as respondents’ self-reported no alcohol drinking or drinking ≤2 alcoholic drinks per day for men and ≤1 alcoholic drink per day for women, and not binge drinking or heavy drinking. Binge drinking was defined as drinking ≥5 drinks on one occasion for men and ≥4 drinks on one occasion for women. Heavy drinking was defined as drinking ≥15 drinks per week for men and ≥8 drinks per week for women in the past 30 days. Having a normal body weight was defined as body mass index ≥18.5 kg/m^2^ and <25 kg/m^2^. Any leisure-time physical activity was defined as an affirmative response to a question, “During the past month, other than your regular job, did you participate in any physical activities or exercises such as running, calisthenics, golf, gardening, or walking for exercise?” Sufficient sleep was defined as ≥7 hours in response to a question, “On average, how many hours of sleep do you get in a 24-hour period?”

The prevalence of performing any leisure-time exercise was higher among cisgender adults (75.5%) than among male-to-female transgender adults (56.7%). More than three quarters (77.4%) of male-to-female transgender adults reported sleeping ≥7 hours during a 24-hour period compared with cisgender adults (65.0%), female-to-male transgender adults (58.9%), and transgender nonconforming adults (52.9%). In addition, male-to-female transgender adults had a lower prevalence of engaging in any two of five health-related behaviors (12.3%) than did cisgender adults (18.6%), but had a higher prevalence of engaging in any three of five health-related behaviors (47.2%) than did female-to-male transgender adults (28.2%) (Table 2).

## Discussion

The findings from this study support those of other studies showing that disparities in sociodemographic characteristics and health-related behaviors exist among the LGBT populations (*3*–*5*). In this study, the disparities were more pronounced among sexual orientation minority adults than they were among transgender adults.

Sociodemographic characteristics and health-related behaviors followed similar yet distinct patterns in the LGBT populations. For example, both home ownership and being married were less prevalent among the LGBT populations than among heterosexual or cisgender adults. However, although results showed that gay men had achieved a higher education level than their heterosexual and bisexual counterparts, this might not necessarily suggest better health-related outcomes or behaviors than those among heterosexual men (*3*). On the other hand, among LGBT populations, bisexual women were found to have higher burdens of health inequalities, which could be related to disadvantaged socioeconomic status, as described in the study findings, or other barriers to health care (*4*). In a study based on 2010 BRFSS data from ten U.S. states, bisexual women were more likely to report fair or poor health status, drink while driving, have asthma, and use equipment for disability, and less likely to seek care owing to cost, than were lesbian women (*6*).

Consistent with findings from previous studies (*3*,*4*), gay men and lesbian and bisexual women were more likely to be current cigarette smokers and were less likely to be moderate drinkers or nondrinkers compared with their heterosexual counterparts. A recent study of media usage by sexual orientation and smoking status found that LGBT adults had more access to Internet and social media than did heterosexual adults (*6*), suggesting that tobacco cessation campaigns could consider multiple educational social media channels to reach out to the LGBT community (*7*).

In this study, lesbian and bisexual women were less likely to report engagement in all five health-related behaviors than were heterosexual women, including being less likely to sleep ≥7 hours during a 24-hour period. Although another study reported no significant difference in sleep duration, the same study noted lesbian and bisexual women were more likely to have poorer quality of sleep with respect to having trouble falling or staying asleep, or taking medication to help sleep than were heterosexual women (*8*). In addition, lesbian women were less likely to have a normal body weight than were heterosexual women. One study found that lesbian and bisexual women were more likely to accept obesity and overweight than were heterosexual women (*9*). Successful intervention studies aiming at reducing overweight and obesity among lesbian and bisexual women have been reported, and more tailored intervention studies are needed to support evidence-based strategies to improve health-related behaviors especially among targeted populations (*10*).

The findings in this report are subject to at least four limitations. First, BRFSS responses are self-reported and, therefore, are subject to reporting and social desirability biases, which might result in underreporting of LGBT status. Second, the findings were limited to 25 U.S. states and Guam and, therefore, might not be generalizable to the entire U.S. population. Third, because of data availability limitations, any leisure-time physical activity was assessed as a single category. Finally, nonresponse bias remains a possibility, although the weighting methodology used by BRFSS adjusts for the nonresponse bias.

Whereas ongoing state-based surveillance data are important to monitor health-related behaviors and outcomes among LGBT populations, the multifaceted causes of health inequality among these populations require further investigation. Continued efforts are needed to plan and implement strategies supported by public health agencies, health care systems, and work sites, as well as targeted strategies in multilevel community-based interventions with social support and educational programs to improve health equity, including engagement in health-related behaviors among LGBT populations.

**Summary**

**What is already known about this topic?**

A higher prevalence of current cigarette smoking and alcohol consumption was observed among U.S. lesbian, gay, and bisexual US adults.

**What is added by this report?**

Compared with heterosexual women (10.6%), the prevalence of not currently smoking cigarettes, moderate or no drinking, maintaining a normal body weight, performing any leisure-time physical activity, and sleeping ≥7 hours per day was lower among lesbian (5.4%) and bisexual women (6.9%). Male-to-female transgender adults had a lower prevalence of engaging in any two of five health-related behaviors (12.3%) than did cisgender adults (18.6%), but had a higher prevalence of engaging in any three of five health-related behaviors (47.2%) than did female-to-male transgender adults (28.2%).

**What are the implications for public health practice?**

Implementation of targeted strategies to increase community-based health intervention programs and mass media campaigns to improve health-related behaviors of lesbian, gay, bisexual, and transgender adults are needed.

## Footnotes

* Deceased.

## Footnotes

¶ https://www.cdc.gov/brfss/index.html.

** Twenty-five states include California, Connecticut, Delaware, Georgia, Hawaii, Idaho, Illinois, Indiana, Iowa, Kentucky, Louisiana, Massachusetts, Minnesota, Mississippi, Missouri, Nevada, New York, Ohio, Pennsylvania, Rhode Island, Texas, Vermont, Virginia, Washington, and Wisconsin.

†† Behavioral Risk Factor Surveillance System 2016 Summary Data Quality Report (https://www.cdc.gov/brfss/annual_data/2016/pdf/2016-sdqr.pdf).

§§ The minimum legal drinking age in the United States is 21 years (https://www.cdc.gov/alcohol/fact-sheets/minimum-legal-drinking-age.htm).

¶¶ Not currently smoking cigarettes was defined as respondents reporting not smoking ≥100 cigarettes during their lifetime or having smoked ≥100 cigarettes during their lifetime but not currently smoking at the time of the survey interview. Moderate or no drinking was defined as respondents reporting no alcohol drinking or drinking ≤2 alcoholic drinks per day for men and ≤1 alcoholic drinks per day for women and respondents reporting no binge drinking and heavy drinking during the 30 days preceding the interview. Binge drinking was defined as ≥5 drinks on one occasion for men and ≥4 drinks for women. Heavy drinking was defined as ≥15 drinks/week for men and ≥8 drinks /week for women during the 30 days preceding the interview. Having a normal body weight was defined as a body mass index ≥18.5 kg/m^2^ and <25 kg/m^2^. Any leisure-time physical activity was defined based on an affirmative answer to a question, “During the past month, other than your regular job, did you participate in any physical activities or exercises, such as running, calisthenics, golf, gardening, or walking for exercise?” Sleeping ≥7 hours during a 24-hour period was calculated based on number of hours respondents answered to a question, “On average, how many hours of sleep do you get in a 24-hour period?” Additional information is available at https://www.cdc.gov/brfss/annual_data/2016/pdf/2016_calculated_variables_version4.pdf and https://www.cdc.gov/brfss/annual_data/2016/pdf/codebook16_llcp.pdf.
